# Supplementary material for: Prediction of ciprofloxacin resistance in hospitalized patients using machine learning
Source: Commun Med (Lond). 2023 Mar 28;3:43. doi: 10.1038/s43856-023-00275-z (PMC10050086; doi:10.1038/s43856-023-00275-z)
Supplement: Supplementary file 1 — Description of Additional Supplementary Files [file 43856_2023_275_MOESM1_ESM.pdf]

Supplementary data titles and legends

Supplementary Data 1: Features that were used and their meaning

Supplementary Data 2: Hyperparameters of the final models. Showing only non-default values.

Supplementary Data 3: Distribution of variable values, stratified by ciprofloxacin resistance. IQR - interquartile range; SD - standard deviation.

Supplementary Data 4: Data used for plotting the resistance trends (figure 1)

Supplementary Data 5: Data used for plotting the ROC curves, calibration plots and net benefit plots (figure 2 and 4).

Supplementary Data 6: Data used for plotting the SHAP plot (figure 3)
