# Supplementary material for: Prediction of ciprofloxacin resistance in hospitalized patients using machine learning
Source: Commun Med (Lond). 2023 Mar 28;3:43. doi: 10.1038/s43856-023-00275-z (PMC10050086; doi:10.1038/s43856-023-00275-z)
Supplement: Supplementary file 8 — Supplementary Information [file 43856_2023_275_MOESM8_ESM.pdf]

Prediction of Ciprofloxacin Resistance in Hospitalized Patients Using Machine Learning

Igor Mintz[1,2], Michal Chowers[3,4], Uri Obolski[1,2,*]

[1]School of Public Health, Tel Aviv University, Tel Aviv, Israel

[2]Porter School of the Environment and Earth Sciences, Tel Aviv University, Tel Aviv, Israel

[3]Meir Medical Center, Kfar Saba, Israel

[4]Sackler School of Medicine, Tel Aviv University, Tel Aviv, Israel

[*]To whom correspondence should be addressed: uriobols@tauex.tau.ac.il

Supplementary material

<u>Figure S1:</u> An illustration of the ensemble model pipeline [1]. L1-LASSO, RF-random forest, XGB-XGBoost, NN-neural network, Y-true resistance

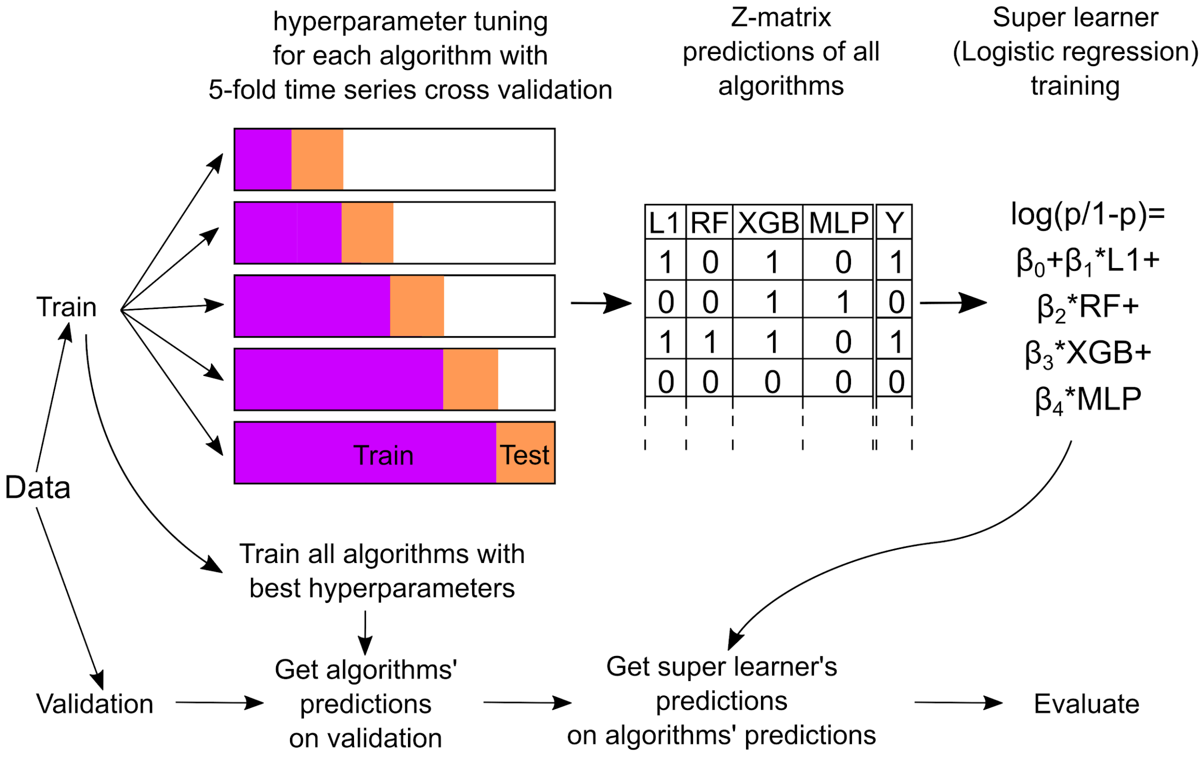

Supplementary reference

1. Van der Laan MJ, Polley EC, Hubbard AE. 2007. Super learner. Stat Appl Genet Mol Biol 6.
